# Low-dose material-specific radiography using monoenergetic photons

**DOI:** 10.1371/journal.pone.0222026

**Published:** 2019-09-06

**Authors:** Joseph Harms, Luke Maloney, Anna Erickson

**Affiliations:** 1 Nuclear and Radiological Engineering and Medical Physics Programs, G. W. Woodruff School of Mechanical Engineering, Georgia Institute of Technology, Atlanta GA 30332, United States of America; 2 Department of Radiation Oncology and Winship Cancer Institute, Emory University, Atlanta GA 30322, United States of America; University of Seville, SPAIN

## Abstract

Cargo containers constitute the most critical component of global trade: 108 million containers represent the movement of about 95% of the world’s manufactured goods. The steady increase in cargo container shipments has had a profound effect on world security: the threat associated with smuggling of shielded special nuclear material is elevated every year. Containers reaching the borders of the U.S. are currently not radiographically inspected due to time and dose considerations stemming from the use of bremsstrahlung beams for imaging. Bremsstrahlung spectra are low-energy peaked, resulting in low penetration values, especially through dense cargoes. The use of monoenergetic radiography beams could alleviate many of these problems due to higher energy and low background continuum. Using Monte Carlo simulations of a realistic imaging scenario with support from previous experimental measurements, we demonstrate how the use of monoenergetic photon beams in radiography can simultaneously reduce the radiation dose imparted to the cargo and any potential stowaways while increasing image quality. Dual-energy methods are leveraged to calculate material atomic number. Image quality is evaluated by measuring the noise standard deviation, contrast-to-noise ratio, and the pixel error as the dose is decreased.

## Introduction

Cargo container shipping accounts for movement of 95% of all manufactured goods internationally [1], moving 4 trillion USD of goods every year [2]. The detection of shielded special nuclear material (SNM) has been named one of engineering’s grand challenges of the 21st century by the Department of Homeland Security [3]. Containers can be probed for presence of SNM by bombarding the cargo with *γ* rays, x rays, or neutrons, for imaging or detection of isotope-specific signatures [4–7]. These active interrogation methods can be more robust than passive interrogation for detection of special nuclear material (SNM), especially in the presence of shielding [8, 9]. However, only around 5% of cargo containers are subject to active interrogation [6].

Implementation of active interrogation is hindered by multiple factors. A primary concern is the radiation dose involved, both to any radiation workers and to the cargo itself, especially in the presence of stowaways. A limit of 500 mrem per scan has been proposed by the National Council on Radiation Protection (NCRP) [10]. Additionally, it has been suggested that an imaging system should take less than 2 minutes to scan an entire container to keep up with throughput requirements imposed at most ports. While multiple vendors produce interrogation systems, the dose and time requirements have proved difficult to satisfy, and these inhibit widespread implementation of active interrogation. In the context of cargo imaging, most systems use bremsstrahlung-generated x-ray sources. These exhibit a continuous energy distribution. While maximum energies up to 9 MeV are available, average energies are typically less than 3 MeV. Many of the low-energy photons in a bremsstrahlung spectrum impart dose to the cargo while never making it to the detection system, increasing the dose necessary to reconstruct high signal-to-noise ratio (SNR) images.

**Fig 1:**
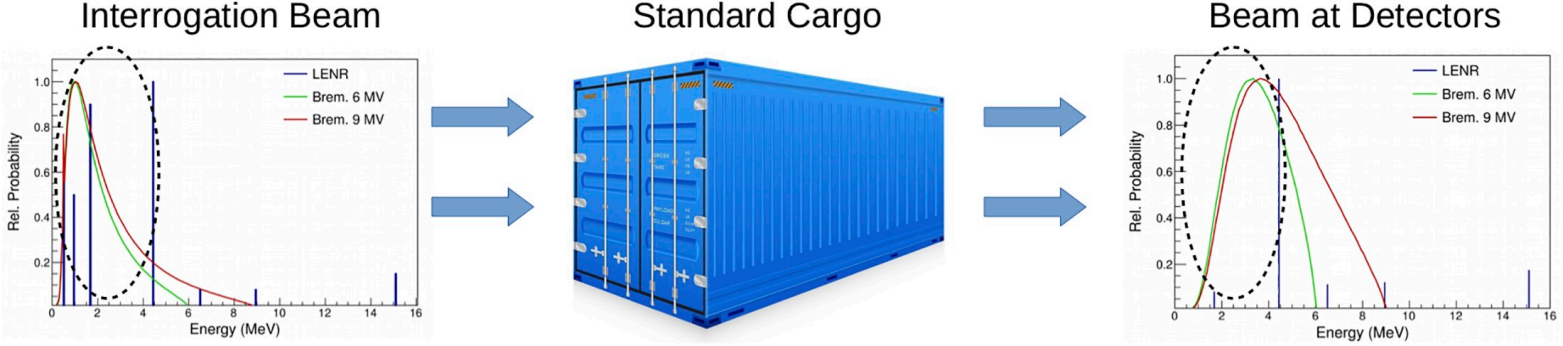
Work-flow of a cargo scanning system from a radiation detection standpoint. The left shows the energy distribution of various interrogation beams and the right shows the energy distribution of those beams after penetration through the container. The black circle highlights the differences in the low-energy end of the spectrum.

If a high-energy monoenergetic beam could be used, each source photon would have relatively high probability of reaching the detector. Such a system would be more efficient, reducing the dose. Fig 1 shows the work-flow of an active interrogation system from a radiation detection standpoint. An interrogation beam bombards the container, and some portion of the beam is stopped in the container, while other components of the beam are transmitted. When using photons as the interrogation source, higher energy sources have stronger penetration. The black circles shown on the spectra showcase the difference in the energy distribution before and after transmission through the container. The low-energy portions of the bremsstrahlung spectra are absorbed by the container, increasing dose to the cargo without contributing information to the detection system. Additionally, the shift in the mean energy of the spectra can lead to inaccuracies when measuring material attenuation.

There are two main methods of producing a high-energy monoenergetic photon source: inverse Compton scattering (also called Thomson scattering) and low-energy nuclear reactions. Inverse Compton scattering produces bursts of high-energy photons by up-scattering optical photons with relativistic electrons [11–14]. Such a system would also allow for pencil beam scanning with higher control of the dose imparted to the cargo. Low-energy nuclear reaction (LENR) beams, such as ^11^*B*(*d*, *nγ*)^12^*C*, can be produced using compact ion accelerators [15, 16]. Previous studies have investigated various LENRs as active interrogation beam sources [17, 18]. In this work, we focus on the ^11^*B*(*d*, *nγ*)^12^*C* reaction, although the results presented here are generalizable to any monoenergetic photon source. The prominent *γ*-ray energies coming from the ^11^*B*(*d*, *nγ*)^12^*C* reaction are at 4.4 and 15.1 MeV, yielding a relatively high-energy beam. The presence of multiple *γ*-ray lines allows for calculation of effective atomic number (Z_*eff*_) via dual-energy methods [19–21]. Previous work has shown feasibility of imaging with a LENR source in a proof-of-concept system [16, 22]. Although we study the implications of monoenergetic photon imaging in reference to cargo screening in this work, the use of monoenergetic photons for imaging can enhance image quality and accuracy in any radiography system and has applications in materials science and characterization, as well as medical and industrial imaging.

A prime advantage of using a nuclear-reaction based beam is that the high- and low-energy images are acquired simultaneously, potentially leading to shorter image acquisition times. Additionally, the images will be perfectly registered, meaning the pixels in both the high- and low-energy images correspond to the exact same point in position and time. Although the pixel values between the two images will be different, their structures will be constant. This redundant structural information can be leveraged to reduce noise on the reconstructed material images. Reducing final image noise allows for a potentially larger reduction in dose as the imaging noise and dose are correlated.

In this paper, we perform Monte Carlo calculations in Geant4 [23] to measure the efficacy of a monoenergetic photon source for imaging. The beam quality metrics tested include penetration, dose to the cargo and detectors, and dose to possible stowaways inside the container. All of these quantities give some indication of the overall imaging performance, and by testing multiple metrics we hope to provide a complete comparison of the capabilities of each beam. Penetration of the beams is simulated through a 20-cm slab of steel, which is used to approximate an average container. Beam transmission is simulated as a function of material and cargo density, building a calibration curve for mapping from transmission to effective atomic number, Z_*eff*_. As a final comparison between bremsstrahlung and LENR-driven beam sources, a full image acquisition is simulated using a phantom containing six different materials, and image quality metrics are evaluated as a function of dose.

## Methods

### Source generation

In this paper, we are comparing bremsstrahlung x-ray beams to a reaction-based *γ*-ray beam (^11^*B*(*d*, *nγ*)^12^*C*) for use as an imaging source. To accurately model the bremsstrahlung source we use the Geant4 Monte Carlo simulation toolkit [23]. In the simulation, electrons are incident on a tungsten target which is 3 mm in diameter. The electrons are sampled from a 2-D Gaussian distribution, with *σ* set to 1.18 mm; the target and source size are taken from reference [24]. A 1.0 cm tungsten filter is placed downstream from the target to filter out low energy x-rays. Multiple filter sizes have been reported in the literature [10, 12, 25], and we chose the tungsten filter to allow for a relatively large amount of filtration. This thickness of tungsten increases the average beam energy from 1.4 MeV with no filtration to 2.0 MeV for the 9 MV beam while maintaining 34% of the flux. The forward-directed x-ray beam is collimated into a cone-shaped geometry with a 2 degree window in the horizontal direction and a 30 degree window vertically, consistent with system parameters reported in reference [12]. Rather than simulating the bremsstrahlung generation process with every simulation, the x-ray energy distribution is collected downstream from the target, filter, and collimator. This energy distribution is then compiled into a probability distribution function and sampled from directly in subsequent simulations, saving the computational cost of electron transport in the target in each simulation. We generate bremsstrahlung spectra with electrons at 6 MeV and 9 MeV.

Although Geant4 is capable of modeling LENRs, we use previously published experimental data to generate our beam source. Measurements were taken at MIT’s Bates Accelerator facility with both LaBr and HPGe detectors, and the details of the experiment and characterization of the beam were carried out by Rose et al [16, 26]. To create the source input for Geant4, an in-house *γ*-ray unfolding code was used to unfold the measured LaBr spectrum [27]. The unfolding code accounts for detector efficiency and can be used to measure the ratio between the *γ*-ray lines. The HPGe data was used to confirm the *γ* ray energies and correct for small errors in the output energies of the unfolding code. In the experimental studies, 35.56 cm (14 in.) of borated polyethylene was used to filter out the neutrons [22], and any leakage neutrons are left out of dose simulations in this paper.

### Penetration and imaging dose

**Fig 2:**
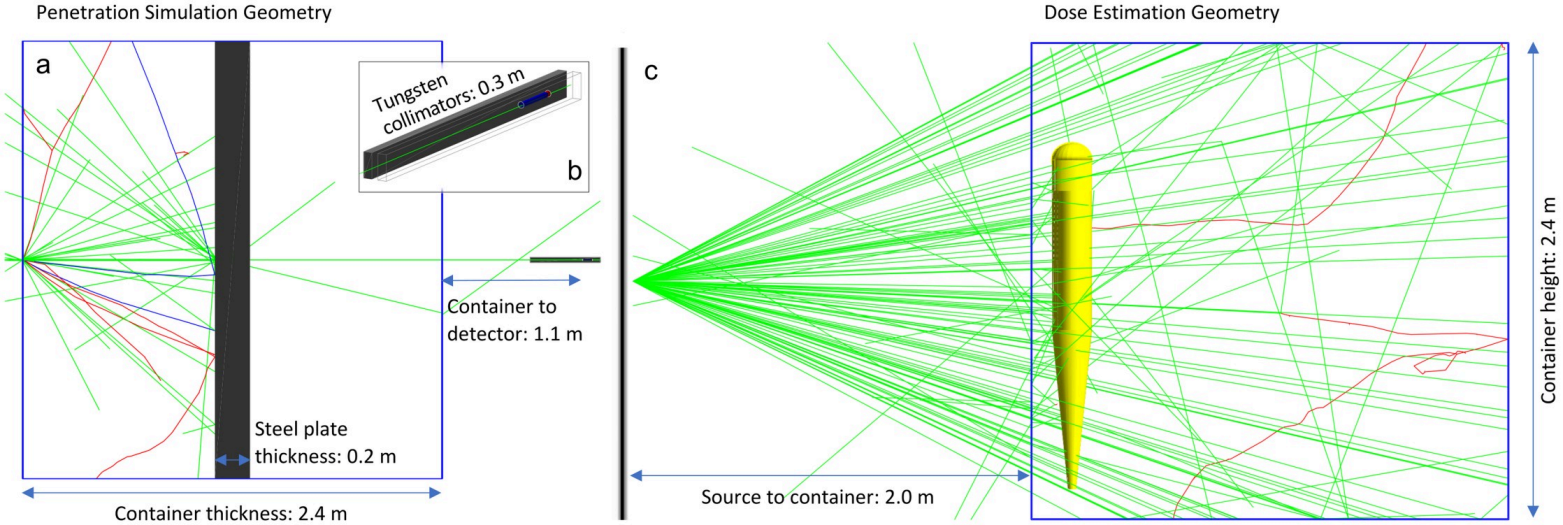
Geant4 geometry for evaluation of detector, cargo, and phantom dose. (a) Photons (green) are incident from left to right, where they enter the cargo container (blue) and penetrate through the steel plate (gray), striking the detector at the right. The source-to-detector distance is just over 3.5 m. (b) is zoomed in on the detector (blue) which is collimated by tungsten plates extending 30 cm in front of the detector face. (c) shows the simulation for dose calculation to a human phantom. This fan-beam geometry was also used to measure dose to the steel plate.

Image noise is inherently dependent on the number of information carriers (i.e. x-rays) which deposit energy in the detector and thus is a function of the beam penetration. We use a Geant4 simulation to characterize the penetration of the radiography beams, with the geometry illustrated in Fig 2. For the penetration simulations, the steel plate thickness was determined based on previous studies which have reported that 80% of cargoes have an average areal density equivalent to 20 cm of steel [10, 19, 28]. The plate is placed in a 2.4 m wide simplified container with 5 mm stainless steel walls. Quartz radiation detectors are placed 1.1 m downstream from the back wall of the container, and dose to the detectors and cargo are tallied as a function of beam type. Vertical tungsten collimators extend 30 cm from the face of the detector to eliminate contributions from scatter in the beam. This simulation geometry matches that presented in reference [12] except that this model uses quartz Cherenkov-based detectors rather than CdWO_4_. For cargo dose measurements, the imaging source was modeled as a fan-beam spanning 60°, as shown in Fig 2c.

To further characterize dose to a stowaway, whole body dose to a numerical human phantom [29] was tallied. The MIRD phantom geometry was copied from the Geant4 human phantom example and placed inside the same container geometry as Fig 2 (with the steel plate removed). It should be noted that for human dose simulations, only the central 60 cm of the container was irradiated, and no cargo besides the human was modeled. This means that the dose to a stowaway could be underestimated at the cost of a more simplified model. However, it is expected that dose from additional cargo will be small relative to primary dose. All simulations were run with at least 8×10^10^ incident photons per beam source. The standard Geant4 physics list FTFP_BERT_HP was used for all simulations so that generation and propagation of any photoneutrons could be tracked.

### Dual-energy transmission

Image quality is not only a function of dose, but of the physical quantity to be measured. Transmission radiography allows for sampling of the linear attenuation coefficient, *μ*. The specific type of interaction a photon undergoes in a material is a function of both the material atomic number and the photon energy. Attenuation coefficient can be broken up into components based on the interaction type, and in the energy domain used for cargo radiography, Compton scattering is the dominant interaction type. However, as the energy and atomic number increase, the probability of pair production increases. In the ^11^*B*(*d*, *nγ*)^12^*C* beam, most of the 4.4 MeV photons will undergo Compton scattering, while a significant amount of 15.1 MeV photons will undergo pair production, especially in high-Z materials. This difference in interaction type can be leveraged to determine atomic number from transmission measurements. In the case of the ^11^*B*(*d*, *nγ*)^12^*C* beam, the transmission of each *γ*-ray is mapped to the attenuation coefficient

$$\begin{array}{c} T\left(E\right)=\frac{I}{I_{0}}=e^{-\mu (E)x} \end{array}$$ (1)

where *I*_0_ is the intensity incident upon the cargo, *I* is the measured intensity after transport through the cargo, *μ*(*E*) is the energy-dependent linear attenuation coefficient, and *x* is the path length of the *γ*-ray through the cargo. The measurement of *μ*(*E*) relies on the use of spectroscopic or photon-counting detectors, as opposed to energy-integrating detectors. If transmission due to separate energies is measured individually, the ratio of their logarithms from Eq 1 becomes independent of *x*:

$$\begin{array}{c} R=\frac{ln(T(H))}{ln(T(L))}=\frac{\mu (H)}{\mu (L)} \end{array}$$ (2)

where *H* and *L* represent high and low energy, respectively. This measured *R* value allows for a mapping from a dual-energy transmission measurement to a single variable which is a function of Z. This formalism can be extended to a dual-energy bremsstrahlung acquisition, but it should be noted that the energy spectrum of a bremsstrahlung beam varies with cargo density, and this will impact the *R* measurement [19, 21]. Since radiography is projection-based imaging, the true measured quantity is termed R_*eff*_. This metric is derivative of Z_*eff*_, a weighted combination of the various materials along a given ray, rather than true material atomic number.

A series of transmission simulations was designed to study the effects of cargo thickness on measurement of *R*-value. A target of variable material and thickness was placed in between a source and a detector. In the calculation of *R*-value, specific regions of each spectrum were integrated. Calculation of the *R*-value relies on integrating certain regions of the detected energy spectrum to measure transmission as a function of energy. The integration regions chosen for the measurement of *R*-values varied between the beam sources. For bremsstrahlung beams, the measurement of *μ*(*E*) can employ either energy-integrating or spectroscopic detectors since the acquisitions can be taken separately. Due to the large amount of spectral overlap between the 6-MV and 9-MV beams, energy-integrating detectors degrade the separation of *μ*(*E*). However, to utilize the energy information of an LENR beam, spectroscopic detectors must be used. Thus spectroscopic detectors were used for both the LENR and bremsstrahlung beams in this work, such that a fair comparison between the systems can be made. For the bremsstrahlung acquisitions, low- and high-energy data were taken separately. The integration regions for each spectrum were empirically tuned to yield the highest change in *R*-value as a function of Z while maintaining adequate statistics. The final energy regions are [1, 5] MeV for the 6-MV bremsstrahlung beam and [5, 9] MeV for the 9-MV bremsstrahlung beam. For the LENR beam, the 4.4 MeV and 15.1 MeV peaks in each spectrum were integrated separately. The use of other lines in the *γ*-ray spectrum, such as the 1.67 MeV line, could yield larger differences in *R* as a function of material. However, no analysis on this was performed here. To keep the results presented in this work generalizable to other systems, the optical physics of the detectors was not simulated, however intrinsic detector efficiency was included in the calculation.

### Imaging simulations

**Fig 3:**
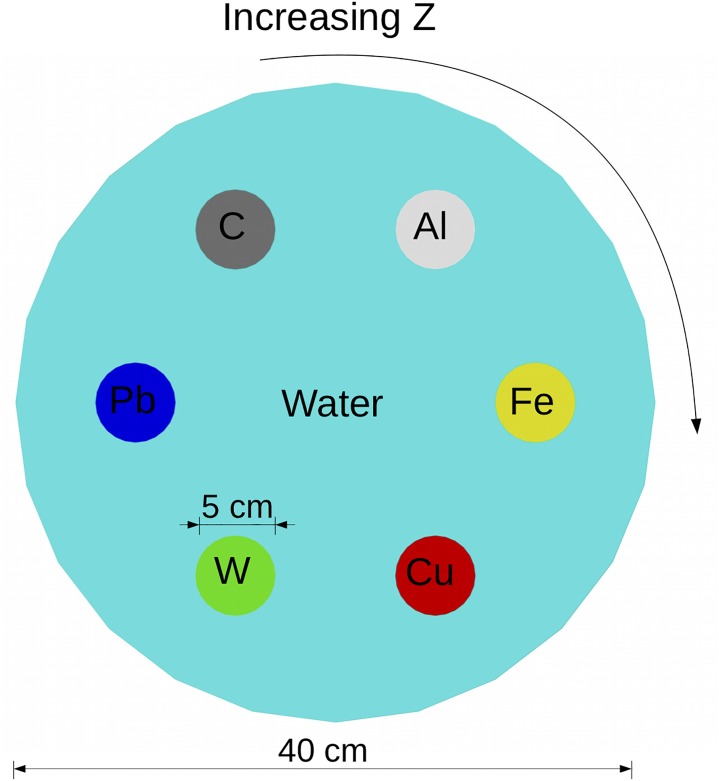
Geant4 phantom used for evaluation of material identification. The elements of each material are shown on the image, and the phantom is suspended in a small-scale cargo container with 5-mm stainless steel walls. The rods increase in Z going clockwise, starting from the carbon.

To test each beam’s material discrimination capability in an imaging scenario, a simple simulation phantom was designed with six cylinders of various materials suspended in a water cylinder as illustrated in Fig 3. Each cylinder is 10 cm thick, and the phantom is placed inside a small-scale cargo container with 5-mm stainless steel walls. To comply with the NCRP goal of keeping cargo screening dose at 500 mrem or less, a dose limit of 5 mrem was set in these simulations. In the simulation, dose is normalized per photon. Since the irradiated area in the simulation is approximately 1/100 the size of the a true container, the smaller dose limit is roughly equivalent to a full scan at 500 mrem.

The material discrimination phantom is imaged by a planar radiation source with width equivalent to the detector diameter (6 mm) and height equal to the detector array height (700 mm). The cargo is stepped in 3 mm increments for a total of 220 steps, leading to a final image size of 660 mm (horizontal) by 700 mm (vertical). The phantom is imaged with each beam separately. The data for the 6 MV brem and 9 MV brem images are combined in post-processing, similar to any dual-energy acquisition system with separate bremsstrahlung imaging beams. For cargo radiography, it has been suggested that adjusting the ratios of low and high energy photons can help minimize dose while increasing information gain. Specifically for the bremsstrahlung beams, the 9-MV provides higher quality information than the 6-MV beam, and vendors have developed uneven dose sharing mechanisms between the two beams in industrial systems. Typically a 1:3 dose sharing ratio between the low- and high-energy beams is used, meaning the low-energy beam delivers 25% of the total imaging dose and the high-energy beam delivers the remaining 75% [21, 30]; this dose sharing ratio was used in this work.

#### Pixel similarity-based noise suppression

The *R*-value calculation described above relies on integrating only small regions of the detected spectra, effectively reducing the detection statistics, and thus the image quality. The information that these discarded photons provide, however, is valuable. While the pixel values between the transmission images (energy-dependent or integrated) and *R*-value images may change, their structures remain constant. Thus, we can use the integrated transmission image, which has lower noise levels than the *R*-value image, to build a material map. The similarity between two given pixels *i* and *k* in a given search window Ω_*i*_ is calculated using an empirical Gaussian model

$$\begin{array}{c} s_{ik}=\{\begin{array}{cc} exp(-\frac{{\left(T\left(i\right)-T\left(k\right)\right)}^{2}}{h^{2}}) & if\;|T\left(i\right)-T\left(k\right)|<3h\;and\;k\in \Omega_{i}\\ 0 & otherwise \end{array} \end{array}$$ (3)

where *s*_*ik*_ is the similarity between pixels *i* and *k*, *T*(*i*) and *T*(*k*) are the measured transmission values at pixels *i* and *k*, and *h* is a user-determined parameter.

This non-local filtration is based on the idea that the true (noiseless) value of an image pixel can be determined by a weighted average of all pixels of the same (or similar) material. Ideally, these similarity values, calculated according to Eq 3, would be calculated for a pixel *i* against all other pixels in the image. However, to improve the efficiency of the algorithm, the pixel similarity is calculated in a neighborhood, Ω_*i*_. The similarity values are stored in a matrix, and they are normalized such that the preserve the signal level of the original image. Noise suppression is then achieved by multiplying the image vector by the similarity matrix.

The strength of the noise suppression is ultimately controlled by two parameters: *h* and the size of Ω_*i*_. A small value of *h* will lead to a tighter similarity window, and may lead to fewer pixels being included in the simulation, while a larger value of *h* may lead to misclassification of materials. A larger Ω_*i*_ will make the noise suppression more non-local, possibly enhancing accuracy at the expense of larger computational times. In this work, *h* is set as the noise standard deviation of the transmission image, and Ω_*i*_ is set to a 31×31 patch, centered about pixel *i*. This form of non-local filtration has been used in image processing, and can be especially useful for noise suppression when prior information is known about the structures within an image [31–33].

#### Image evaluation

For quantitative evaluation of imaging performance, we measure pixel error, contrast-to-noise ratio (CNR), and image noise. Pixel error is measured as

$$\begin{array}{c} E_{mat}=\frac{|R_{mat}-R_{NIST}|}{R_{NIST}} \end{array}$$ (4)

where *R*_*mat*_ is the measured *R*-value of a given material, *R*_*NIST*_ is the expected *R*-value based on data from NIST’s XCOM database [34]. CNR is measured as

$$\begin{array}{c} CNR_{mat}=\frac{|R_{mat}-R_{water}|}{\sigma_{water}} \end{array}$$ (5)

where *σ*_*water*_ is the noise standard deviation of a uniform region of the image. This is calculated over 1650 pixels in a central region of the phantom to give an accurate estimate of the total image noise standard deviation (SD). Noise SD measures the pixel-to-pixel variation and can account for the graininess present in an image. For all imaging simulations, the dose to the cargo between the combined bremsstrahlung acquisition and the LENR acquisition is matched.

All simulations were carried out in Geant4, data analysis and image processing were done in the open source analysis software ROOT [35], and the noise suppression algorithm is implemented in Matlab.

## Results

### Beam penetration and imaging dose

The LENR beam produced 2.7 times more signal in the detector per particle after penetrating through the 20 cm steel plate, as shown in Table 1. This means that fewer particles will be needed to create similar image quality in a full-scale system than with bremsstrahlung, lowering the dose delivered to the cargo and any potential stowaways. Table 1 also shows the plate and phantom dose simulation results. The LENR beam delivers 1.6 times more dose per source photon to the plate and 1.4 times more dose to the phantom than the 6 MV bremsstrahlung beam, also shown in Table 1. When combining the increased light output with the human dose, the LENR method provides a 40% reduction in dose to the human phantom, as compared to the 6-MV bremsstrahlung beam, to produce the same signal in the detector. In Table 1, the relative light output correlates to detector dose while accounting for the Cherenkov threshold of quartz. The final column shows the scaled relative dose to the cargo, which accounts for the 1:3 dose sharing ratio of the bremsstrahlung spectra and their effective doses per source particle.

**Table 1:** Results from beam penetration simulations using the LENR beam and both bremsstrahlung beams.

Beam	Penetration (%)	Relative Light Output	Plate Dose (mrem/photon)	Phantom Dose (mrem/photon)
6*MVBrem*.	0.11	1.00	4.31E-10	4.72E-9
9*MVBrem*.	0.17	1.85	5.53E-10	5.59E-9
*LENR*	0.26	2.69	6.90E-10	6.36E-9

### Dual-energy transmission

**Fig 4:**
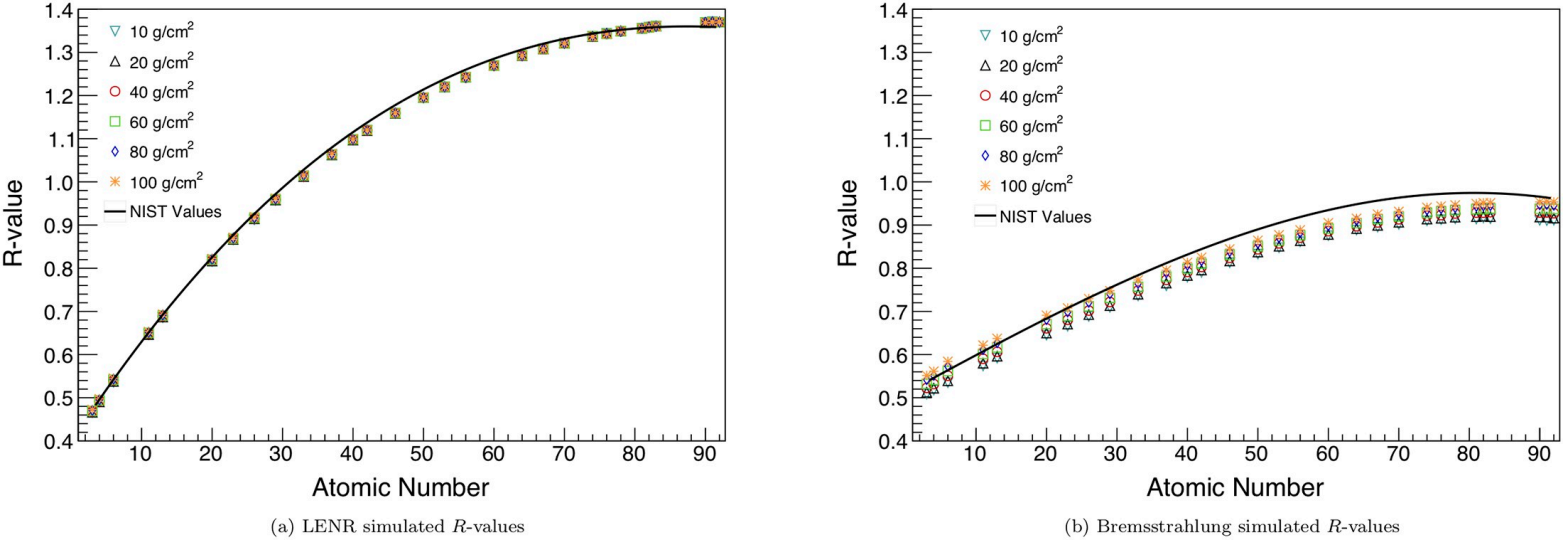
Output *R*-values from the transmission simulation for the ^11^*B*(*d*, *nγ*)^12^*C* beam (a) and the bremsstrahlung beams (b). A fit to the values on NIST XCOM for the mean beam energies is also shown on each plot [34]. The range of *R*-values is higher for the LENR beam, and the simulated values are more accurate to the NIST data. Additionally, the *R*-values from the LENR beam are independent of cargo thickness, while they tend to increase with thicker cargo for the bremsstrahlung beams. Error bars are shown on the plot, though they may be smaller than the data markers.

The LENR beam produces the same *R*-value for a given material at all target thicknesses tested, while the bremsstrahlung acquisition gives an *R*-value which increases with cargo areal density. This highlights a key advantage of monoenergetic beams; as cargo thickness is increased, the effective energy of the high and low energy regions does not change. However, for continuous spectra, the low energy photons are preferentially filtered out by the cargo, and thus the mean beam energy increases with cargo thickness, changing the effective linear attenuation coefficient, *μ*_*eff*_. Additionally, the *R*-value varies over a wider domain when measured with the LENR beam than with the bremsstrahlung beams, as shown in Fig 4. This is due to the larger pair production interaction probability at 15.1 MeV than at 6 MeV, the average energy of the bremsstrahlung high energy region.

### Low-dose imaging and material identification

**Fig 5:**
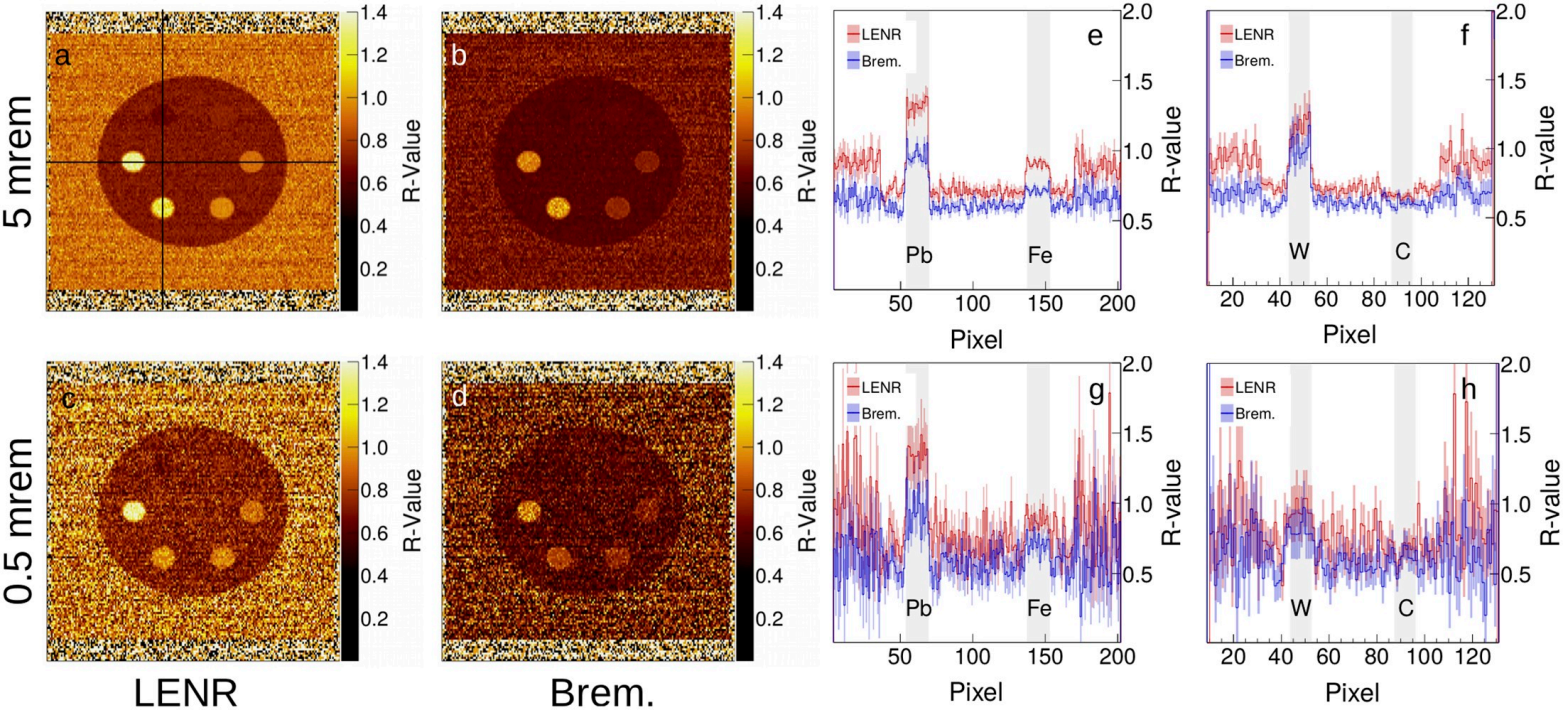
(a)-(d) Reconstructed *R*-value images and (e)-(h) the corresponding line profiles in the horizontal and vertical directions.

At full dose, all six rods can be seen in both LENR- and bremsstrahlung-generated images, however the difference between the rods stands out more with the LENR image, as seen in Fig 5a–5d. This is because of the larger range of *R*-values achievable with the LENR beam, as discussed previously. As the dose is reduced, higher contrast present on the LENR image allow all six rods to remain partially visible while the low-Z rods (carbon and aluminum) are lost on the bremsstrahlung image. Additionally, the difference between the lead and tungsten is larger in the LENR images. Fig 5e–5h show the line profiles in the horizontal and vertical directions. (e) and (g) show the horizontal line profile through the lead and iron rods in the high and low dose images, respectively. At high dose, both rods are clearly visible, while at low dose, the iron gets lost in the noise. (f) and (h) show a profile through the tungsten and carbon rods for high and low dose images, respectively. In this more challenging case, the carbon is relatively difficult to see, even at high dose. Both rods tend to be buried in the noise at low dose.

The error and contrast for the images are shown in Table 2. The LENR method outperforms the bremsstrahlung method in CNR by 50% and 75% for the high and low dose images, respectively, for the same cargo dose. CNR dictates how detectable an object is in both human and computer vision [36–38]. In the context of cargo scanning, higher CNR will increase the true positive rate by allowing high-Z objects to stand out. Additionally, the false positive rate can be reduced because of decreased error margins on the threshold for a threatening object.

**Table 2:** Contrast-to-noise ratio and pixel error for each material in images (b)-(e) shown in Fig 5. The final row shows the root-mean-square error.

	CNR				Error			
	LENR		Brem.		LENR		Brem.	
Z	5 mrem	0.5 mrem	5 mrem	0.5 mrem	5 mrem	0.5 mrem	5 mrem	0.5 mrem
6	1.64	0.67	0.41	0.10	1.91	2.06	3.69	3.28
13	0.78	0.22	0.79	0.13	1.75	0.02	2.89	5.09
26	5.64	1.64	3.37	1.11	1.37	1.46	1.74	0.94
29	7.03	2.11	4.08	1.41	0.47	0.27	1.28	0.56
74	13.68	2.36	11.97	1.89	10.61	33.77	4.77	18.85
82	17.75	5.41	10.35	3.21	0.98	0.22	0.37	0.75
*RMSE*	7.75	2.07	5.16	1.31	4.52	13.82	2.87	8.10

The two beams had similar performance with respect to error, except for the tungsten rod, where the LENR beam produced about twice as much error as the bremsstrahlung beams. The larger error in the tungsten can be accounted for by a much higher pair production cross section for the 15.1 MeV *γ*-ray than with the bremsstrahlung beams. The relatively high areal density of 193 g/cm^2^ means that very few 15.1 MeV photons can penetrate through the materials, and this is exacerbated at low dose.

**Fig 6:**
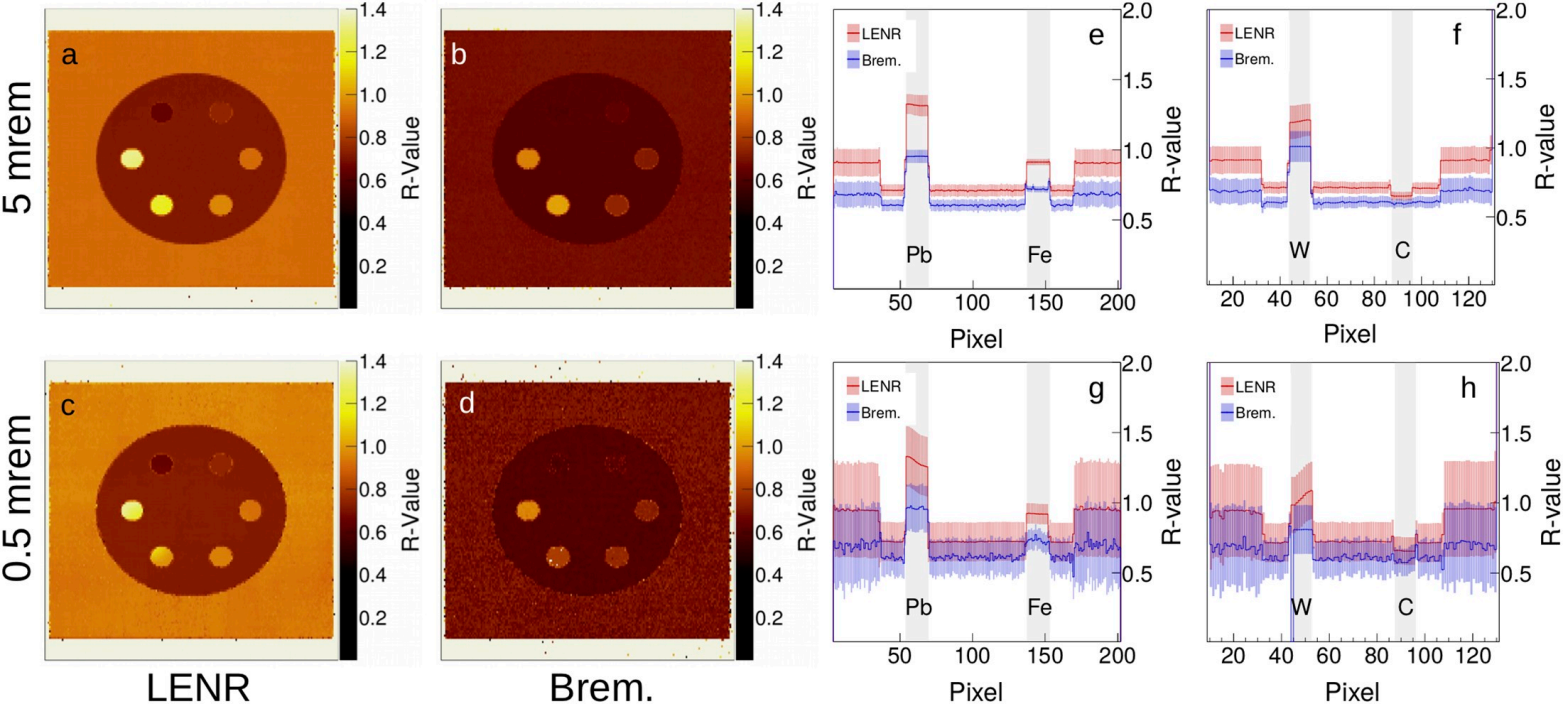
(a)-(d) Noise suppressed *R*-value images and (e)-(h) their corresponding line profiles.

The noise-suppressed *R*-value images and their cross line profiles are shown in Fig 6a–6d. The noise suppression method is very effective for both bremsstrahlung and LENR acquisitions. At low dose, the noise suppression is stronger for the LENR image. Additionally, because of the lower inherent contrast in the bremsstrahlung images, the carbon rod is largely missed in the pixel similarity calculation, as shown specifically by Fig 6f. At lower dose, an artifact can be seen on the tungsten and lead rods. The pixel values are pulled closely together as the spacing between given pixels decreases, and this leads to the slanting line profiles on the rods seen in Fig 6g and 6h.

**Fig 7:**
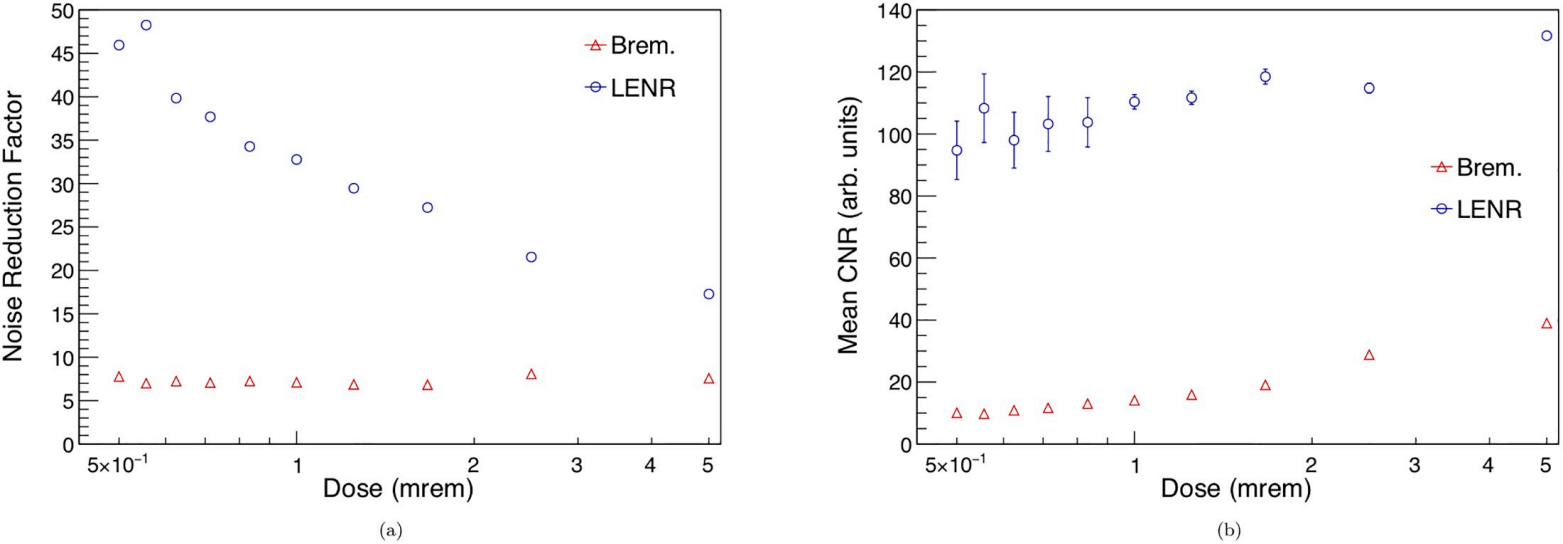
(a) The noise reduction factor for both bremsstrahlung and LENR generated images as a function of dose is shown in the left. As the dose gets lower, noise suppression gets stronger for the LENR beam, keeping the image noise level relatively constant. (b) The mean contrast-to-noise ratio for the six rods in each noise-suppressed image, as function of dose. Although the CNR for the LENR does decrease with dose, it remains around a factor of 10 higher than the CNR achieved with the bremsstrahlung imaging source.

Fig 7 shows the noise reduction and mean CNR on the noise suppressed images as a function of imaging dose. Noise reduction factor is simply *σ*_*noisy*_/*σ*_*suppressed*_, and the noise suppression gets stronger for the LENR image as dose is decreased, leading to similar levels of imaging noise on the final image. The bremsstrahlung noise suppression, however, is relatively constant, leading to reduced efficacy of the algorithm at lower dose. This is due to the higher inherent contrast levels of the low-energy nuclear reaction imaging source. As dose decreases, image noise grows, and low contrast objects are lost in the noise, as shown in Fig 6e–6h. After noise reduction, the LENR images show a 358% and 941% improvement in CNR over the bremsstrahlung images, while adding little error to the original image, as shown in Table 3. This large improvement in CNR can lead to much more sensitive detection of small objects and potential threats in the context of cargo scanning, making the LENR source more reliable for imaging.

**Table 3:** Contrast-to-noise ratio and pixel error for each material in images (a)-(d) shown in Fig 6.

	CNR				Error			
	LENR		Brem.		LENR		Brem.	
Z	5 mrem	0.5 mrem	5 mrem	0.5 mrem	5 mrem	0.5 mrem	5 mrem	0.5 mrem
6	29.12	26.63	2.24	0.03	2.27	1.24	2.99	1.25
13	14.58	10.40	6.51	1.83	1.55	0.63	2.46	3.07
26	97.97	81.41	25.64	8.91	1.32	0.45	1.61	0.36
29	121.63	93.83	30.73	10.84	0.53	1.65	1.35	0.39
74	232.95	124.24	90.73	13.16	11.35	29.39	4.81	21.97
82	293.85	231.77	78.12	25.66	1.07	3.37	0.47	0.62
*Mean*	131.68	94.71	39.00	10.07	4.82	12.11	2.67	9.07

## Discussion

The monoenergetic source tested in this work is driven by a low-energy nuclear reaction, namely ^11^*B*(*d*, *nγ*)^12^*C*. Reaction-based sources produce near-isotropic radiation beams which have to be collimated down to a fan beam, wasting much of the flux. It is possible that collimation of these beams into multiple views could yield near-tomographic images, although little work on this has been done.

Reaction-based sources can produce multiple monoenergetic *γ*-rays simultaneously, leading to perfect image registration and material discrimination, given the *γ*-ray lines are well-separated in energy and spectral detectors are used. Although the energies of a given reaction cannot be altered, there are multiple reactions to choose from, which allows for some control over the system. For example, ^12^*C*(*p*, *p*′*γ*)^12^*C* can produce the same states of carbon as ^11^*B*(*d*, *nγ*)^12^*C*, but without generation of neutrons. If it was desirable to keep photon energies under 10 MeV to avoid photoneutron production, ^16^*O*(*p*, *p*′*γ*)^16^*O* produces *γ*-rays with maximum energy at 7.12 MeV [39]. It should also be noted that while these sources show promise for imaging purposes, the flux produced by most particle accelerators today is insufficient for imaging purposes, especially if the Department of Homeland Security goal of scanning in under two minutes is to be met.

The beam source used affects the output image quality, but the detector array also has an impact on the output image quality. Detectors for active interrogation imaging applications are typically tightly packed into arrays, the geometry of which can affect both the spatial resolution and the quality of the signal [40]. For increased signal-to-noise ratio (leading to potential reductions in dose), the detectors can be synchronized with certain sources, for example, Inverse Compton scattering (ICS) sources. ICS sources can produce nearly monoenergetic photons in a pulse-type mode which would allow for greatly reduced scatter if the detectors only read out after trigger pulses from the accelerator. Additionally, these sources offer a tunable energy and output, which could be ideal for modulating dose based on cargo density. These sources have small emission spot sizes and allow for tight control over the angular spread of the photons, leading to potentially greater dose reductions than those which are shown in this work. Much like reaction-driven sources, ICS-driven beams are under development and higher fluxes are needed before implementation.

The *R*-value reconstruction, which maps to material atomic number, is energy-dependent. If the high-energy bremsstrahlung beam instead had an end point of 15 MeV, it would achieve a higher-range *R*-value curve, although it would not be as strong as the LENR beam. Additionally, if a LENR that produced lower-energy *γ* rays were used, the *R*-value contrast would not be as high as what is shown here.

The LENR method produces inherently higher-contrast images than bremsstrahlung, the industry standard. This leads to better detection of potentially smuggled special nuclear materials, especially when the imaging dose is reduced. Additionally, the LENR beam allows for simultaneous acquisition of low and high energy images, allowing for enhanced noise suppression techniques based on redundant structural information. On average, the LENR method outperforms bremsstrahlung produced images in CNR by a factor of 7.5 over all imaging doses tested. At the lowest dose tested, corresponding to roughly 1/10 of the dose limits for cargo radiography, the LENR images showed a higher average CNR by a factor of 9.4. CNR is an important metric as it can dictate how detectable an object is in both human and computer vision [36–38]. A higher CNR means that a given object will stand out against the background stronger. In the context of cargo scanning, this is important because potential threats which stand out above the background level are more likely to be detected, increasing the true positive rate. Additionally, if an image has higher inherent CNR, the false positive rate can be reduced because the error margin on the threshold for a threatening object can be reduced. The noise suppression was more effective on the LENR images, consistently reducing noise to the same levels even as the dose was decreased. This could be extrapolated to show that similar or superior image quality with the LENR beam can be achieved at lower doses when noise suppression is used. This paper shows that using a nuclear-reaction based imaging beam can improve the efficacy of cargo radiography compared against the industry standard, while maintaining or decreasing the radiation dose.

## Conclusion

We have shown that the use of a monoenergetic photon source, specifically the ^11^*B*(*d*, *nγ*)^12^*C* reaction, can decrease the radiation dose used to acquire an image while increasing the contrast-to-noise ratio of the image. We have tested this method in the application space of cargo screening, but the use of monoenergetic photon imaging has potential applications in medicine and materials science.

Bremsstrahlung beams are the industry standard for imaging, in large part because of their relative ease of production and the high photon fluxes available. However, bremsstrahlung beams exhibit a low-energy peaked continuous energy distribution. This leads to lower beam penetration and less efficient use of the imaging dose because more incident photons are required to produce similar detectable signals. This work shows that use of monoenergetic photons for imaging increases penetration as well as material specificity.
